# Risk of Congenital Toxoplasmosis in Newborns from Mothers with Documented Infection: Experience from Two Referral Centres

**DOI:** 10.3390/pathogens14020157

**Published:** 2025-02-06

**Authors:** Alice Bonetti, Agnese Comelli, Annacarla Chiesa, Vania Spinoni, Ambra Vola, Federico Prefumo, Adriana Valcamonico, Carlo Bonfanti, Silvio Caligaris, Lina Rachele Tomasoni, Fausto Baldanti, Valeria Meroni

**Affiliations:** 1 Microbiology and Virology Unit, Diagnostic Medicine Department, Fondazione IRCCS Policlinico San Matteo, 27100 Pavia, Italy; 2 PhD National Programme in One Health Approaches to Infectious Diseases and Life Science Research, Department of Public Health, Experimental and Forensic Medicine, University of Pavia, 27100 Pavia, Italy; 3 Infectious Diseases Unit, Foundation IRCCS Ca’ Granda Ospedale Maggiore Policlinico, 20122 Milan, Italy; 4 Department of Infectious and Tropical Diseases, ASST Spedali Civili, 25123 Brescia, Italy; 5 Department of Infectious Diseases, Manzoni Hospital, 23900 Lecco, Italy; 6 Department of Neonatology and Neonatal Intensive Care, Spedali Civili Hospital, Spedali Civili, 25123 Brescia, Italy; 7 Obstetrics and Gynecology Unit, IRCCS Istituto Giannina Gaslini, 16147 Genova, Italy; 8 Department of Obstetrics and Gynecology, University of Brescia, 25123 Brescia, Italy; 9 Department of Molecular and Translational Medicine, Institute of Microbiology, University of Brescia-ASST Spedali Civili, 25123 Brescia, Italy; 10 Dipartimento di Scienze Clinico-Chirurgiche, Diagnostiche e Pediatriche Università di Pavia, 27100 Pavia, Italy; 11 Department of Molecular Medicine, University of Pavia, 27100 Pavia, Italy

**Keywords:** toxoplasmosis in pregnancy, *Toxoplasma gondii*, amniocentesis, spiramycin, pyrimethamine, sulfadiazine

## Abstract

During pregnancy, primary *Toxoplasma gondii* infection can cause congenital toxoplasmosis (CT). We described the newborns’ outcomes from a multicentre cohort of mothers with seroconversion (SC) at different gestational ages. This retrospective observational study (from 2007 to 2018) was conducted in two Italian referral hospitals: Fondazione IRCCS Policlinico San Matteo in Pavia and Spedali Civili in Brescia. In total, 247 pregnant women were enrolled: seroconversions were enrolled: seroconversions documented as having occurred in the two months preceding pregnancy in 12 cases (4.9%; 95% CI 2.2–7.5%), and during pregnancy in 235 cases (95.1%; 95% CI 92.5–97.8%). SC is defined as the appearance of specific anti-Toxoplasma antibodies (IgM/IgG) during pregnancy in a previously seronegative woman. A total of 56 (22.5%; 95% CI 17.3–27.7%) newborns were lost to follow-up; thus, the outcome of 193 (77.5%; 95% CI 72.3–82.7%) newborns was analyzed. The overall transmission rate of *T. gondii* infection was 23.8% (95% CI 17.8–29.8%), 0% (95% CI 0.0–11.9%) among the 1st trimester SCs, 12.5% (95% CI 5.6–19.4%) among the 2nd trimester SCs, 53.8% (95% CI 41.7–66.0%) among the 3rd trimester ones. No CT were found in the group of periconceptional infection. Among the infected newborns, clinically manifest cases were 12 (26.1%; 95% CI 13.4–38.8%), including 1 case (2.2%; 95% CI 2.0–6.4%) of stillbirth and 11 symptomatic neonates (23.9%; 95% CI 11.6–36.2%). A total of 83 amniocentesis were performed (33.6%; 95% CI 27.7–39.5%), no complication was recorded and no false positive or false negative results were registered. The results are in line with the fetal risks reported in literature for *T. gondii* infection during pregnancy, even if at a lower percentage probably due to a prompt treatment.

## 1. Introduction

Toxoplasmosis is a systemic infection caused by *Toxoplasma gondii*, a protozoan belonging to the phylum Apicomplexa. *T. gondii* is capable of infecting a wide range of hosts, including birds and aquatic and terrestrial mammals, including humans. In addition to that, oocysts of *T. gondii* are harboured in bivalve molluscs. In immunocompetent hosts, the infection is usually asymptomatic and self-limiting, controlled by both cell-mediated and humoral immunity [1].

Congenital toxoplasmosis (CT) is the consequence of *T. gondii* vertical transmission from mother to fetus after a primary *T. gondii* infection during pregnancy. Seroconversion (SC) is the definitive serological evidence of first-time infection, that is, the appearance of specific anti-Toxoplasma antibodies (IgM/IgG) during pregnancy in a previously seronegative woman.

Another at-risk scenario for pregnancy is represented by periconceptional infections (occurring within 2 months prior to the last reported menstrual period and up to 3 weeks following the expected date of the missed menstrual period). This clinical picture is defined by a low or intermediate anti-Toxoplasma IgG avidity index in the first trimester of pregnancy [1].

According to the literature, the overall transmission rate increases with the gestational age at which the infection occurs, while the severity of disease in the fetus is inversely proportional to the time of maternal infection [1,2].

According to a recent French study, the percentages of maternal–fetal transmission at 6, 18, and 30 gestational weeks are, respectively, 2.2%, 23.0%, and 56.0%. The French group conducted a literature review and analyzed the databases of Paris, Lyon, and Marseille regarding 5000 cases of maternal seroconversions, with respective neonatal follow-up [3].

A recent Italian study showed a very low risk (0.13%) of vertical transmission of *T. gondii* in women receiving spiramycin throughout pregnancy following a IgM+/IgG+/low–intermediate IgG avidity index in the first trimester without documented seroconversion [4].

The consequences of fetal infection vary, including spontaneous abortion, stillbirth, permanent neurological sequelae or visual impairment, and completely asymptomatic infections [1,2]. Approximately 20% of infants with CT have clinically apparent signs and symptoms at birth or in early infancy [5].

The above-mentioned figures on transmission and CT risk are crucial during a consultation for *T. gondii* infection in pregnancy to inform the patient of the possible neonatal outcomes based on the timing of infection and the therapeutic management [5,6]. The aim of this retrospective observational multicentre study was to describe the outcomes of newborns from mothers with documented SC or periconceptional infection from a multicentre cohort in two tertiary referral hospitals in Italy: Fondazione IRCCS Policlinico San Matteo in Pavia and Spedali Civili in Brescia. Another objective of our study was to identify possible gaps in the clinical management of these pregnancies.

## 2. Materials and Methods

This retrospective observational study was conducted in two Italian referral hospitals: Fondazione IRCCS Policlinico San Matteo in Pavia and Spedali Civili in Brescia.

In both centres, there is a dedicated multidisciplinary team of infectious disease or microbiology specialists, gynecologists who are experts in ultrasound diagnosis of pregnancy infections, and neonatologists. They are responsible for diagnosis, counselling, and follow-up.

Data were collected from 2007 to 2018, including all patients with confirmed *T. gondii* periconceptional infections or SC during pregnancy.

Cases of voluntary termination of pregnancy (ToP) were excluded from the analysis.

For serological diagnosis, the Microbiology and Virology laboratory in Brescia used IgG, IgM, and IgG avidity tests (BEIA kit; Bouty, Milan, Italy). In the Microbiology and Virology laboratory in Pavia, routine tests included IgG and IgM (Diasorin, Saluggia, Italy), along with IgG ELFA. IgM ISAGA, IgG Avidity (Biomérieux, Marcy l’Étoile, France), IgA ELISA (Diasorin), and IgG/IgM immunoblot (IB—Toxoplasma Western blot IgG/IgM; LDBIO, Lyon, France) were employed as second-line tests in cases of discordant or inconclusive results.

According to the Italian guidelines for toxoplasmosis in pregnancy, patients were treated with spiramycin 3 MU (million units) three times a day until delivery. Alternatively, in the case of late seroconversion (after the 21st week of pregnancy), patients were treated with pyrimethamine-sulfadiazine plus folinic acid.

Another scenario for treatment with pyrimethamine-sulfadiazine was a positive PCR for *T. gondii* on amniotic fluid; in this case, spiramycin was suspended, pyrimethamine-sulfadiazine prescribed, and spiramycin alone restarted about three weeks before childbirth [7,8,9,10].

Ultrasound scans were routinely scheduled every 4–6 weeks until delivery. Moreover, amniocentesis was proposed starting from 18 weeks of gestation, and at least 4–6 weeks after infection. The amniotic fluid underwent double testing for *T. gondii* DNA utilizing real-time PCR Toxoplasma g ELITe MGB (ELITech Group SPA, Turin, Italy) with a rep529-targeting assay, post-extraction via easyMAG (Biomérieux Marcy l’Étoile, France) [11,12,13].

Concerning the newborns’ follow-up, all neonates at risk for congenital toxoplasmosis (confirmed maternal infection, with or without prenatal diagnosis) underwent a full clinical and neurological assessment at birth, specific seroimmunological tests, direct and indirect dilated fundoscopy (to detect chorioretinitis or other related ocular conditions), and transfontanellar ultrasound (to exclude ventricular dilatation, cerebral calcifications, or porencephaly). Serological monitoring was advised until the child reached 1 year of age and included anti-*T. gondii* IgG, IgA, and IgM via immunoassay and IgG/IgM immunoblot (IB), with a comparison of their immunological profiles to those of their mothers at birth and then with the newborn samples at birth in the first two months of life [3,7].

Serological tests were conducted monthly during the first three months, and then every two months until one year of age. The absence of congenital infection was confirmed by the negativization of specific antibodies within the first year of life in the absence of therapy. Conversely, an increase in IgG levels or the emergence of IgM and/or IgA antibodies indicates congenital infection.

The diagnosis of congenital toxoplasmosis was established in the following cases:

- Positive IgM or IgA for *Toxoplasma gondii*;
- IgG/IgM synthesized by newborn, identified through comparative IB (ImmunoBlot);
- IgG rebound and/or persistence at 12 months of age.

In the absence of congenital infection, the last clinical examination and fundus evaluation were performed at one year of age. However, in cases of congenital infection, long-term ophthalmological, neurological, and auditory follow-up were performed.

For each pregnancy, serological, clinical, therapeutic data and newborn outcomes were recorded.

Statistical analyses were performed using Stata (version 15). Descriptive statistics, including counts and proportions, were computed for all variables. Group comparisons were made using chi-square or Fisher’s exact test as appropriate. Two-tailed tests were employed, with statistical significance set at *p*-values < 0.05.

The study obtained ethical approval from both centres: Pavia in March 2023 (protocol number 0011846/23) and Brescia in May 2024 (protocol number 0025614/24).

## 3. Results

A total of 247 pregnant women were enrolled in this study: seroconversions documented as having occurred in the two months preceding pregnancy in 12 cases (4.9%; 95% CI 2.2–7.5%) and during pregnancy in 235 cases (95.1%; 95% CI 92.5–97.8%). Our cohort included three (1.2%; 95% CI 0.2–2.6%) twin pregnancies, and we excluded from the analysis one case of voluntary ToP (0.4%; 95% CI 0.0–1.2%) reported in a patient with a confirmed seroconversion at 7 weeks of pregnancy. Thus, the total number of newborns included in the analysis was 249. Table 1 summarizes the results of this study.

The study population consisted of women aged between 27 and 34 years (median: 30.5 years). The majority of patients were Italian, 177 (71.6%; 95% CI: 66.0–77.3%), while 58 were born abroad (23.5%; 95% CI: 18.2–28.8%). Information on the country of origin was not available for 12 women (4.9%; 95% CI: 2.2–7.5%).

Out of the 235 documented seroconversions, 38 (16.2%; 95% CI: 11.5–20.9%) were in the first trimester, 122 (51.9%; 95% CI: 45.5–58.3%) in the second, and 75 (31.9%; 95% CI: 26.0–37.9%) in the third.

Three twin pregnancies occurred in the group of SC in the second trimester; thus, 125 newborns were evaluated in that group.

Spiramycin was the first-choice therapy in 223 pregnant women (90.3%; 95% CI: 86.6–94.0%), interrupted only in three cases (1.3%; 95% CI: 0.0–2.9%) due to side effects. For these three patients, an intermittent protocol was adopted: one week of spiramycin therapy and one week of therapy suspension, until delivery. Fifty-one patients (20.6%; 95% CI: 15.6–25.7%) were treated with pyrimethamine-sulfadiazine plus folinic acid, both for pregnant women for whom pyrimethamine-sulfadiazine was the first therapeutic choice (late seroconversion) and for cases of therapeutic shift from spiramycin to pyrimethamine-sulfadiazine (positive amniocentesis).

In nine cases (17.6%; 95% CI: 7.2–28.1%), pyrimethamine-sulfadiazine therapy was discontinued due to side effects, and treatment with spiramycin was resumed. For four patients (1.6%; 95% CI: 0.0–3.2%), we have no information regarding therapy, and 6 women (2.4%; 95% CI: 0.5–4.3%) did not receive any treatment.

A total of 83 amniocenteses were performed (33.6%; 95% CI: 27.7–39.5%), 70 during the second trimester (84.3%; 95% CI: 76.5–92.2%) and 13 during the third trimester (15.7%; 95% CI: 7.8–23.5%). Only 5 out of the 83 PCRs on amniotic fluid tested positive for *T. gondii* (6.1%; 95% CI: 0.9–11.1%). All five positive results were confirmed as congenital infections by serological exams after birth.

No complications occurred following amniocentesis, and no false positive or false negative results were observed. All women undergoing amniocentesis were under treatment at the time of the procedure.

A total of 56/249 (22.5%; 95% CI: 17.3–27.7%) newborns were lost to follow-up; thus, the remaining 193 (77.5%; 95% CI: 72.3–82.7%) newborns were analyzed. Between newborns lost to follow-up, 9/56 (16.0%; 95% CI: 6.5–25.7%) were in the group of first-trimester SCs, 37/56 (66.1%; 95% CI: 53.7–78.5%) in the group of second-trimester SCs, and 10/56 (17.9%; 95% CI: 7.8–27.9%) in the group of third-trimester SCs.

The vertical transmission of *T. gondii* infection occurred in 46 out of 193 newborns, with an overall transmission rate of 23.8% (95% CI: 17.8–29.8%).

**Figure 1:**
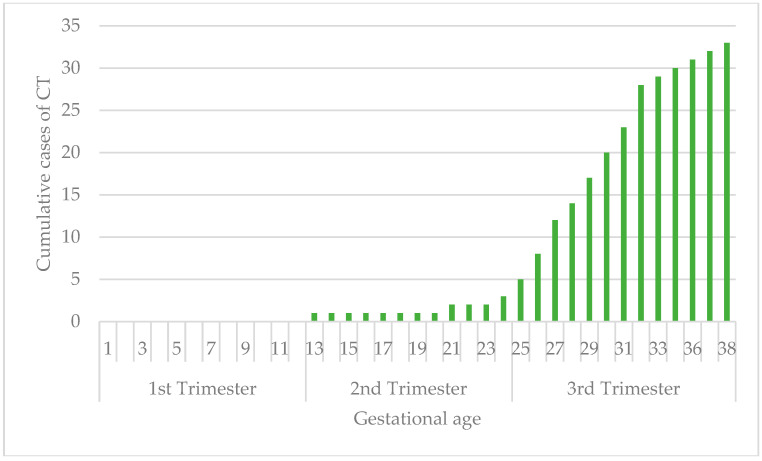
Vertical transmission rate related to time of maternal infection.

The rate of vertical transmission is related to maternal gestational age: 0/29 (0%; 95% CI: 0.0–11.9%) in the first trimester, 11/88 (12.5%; 95% CI: 5.6–19.4%) in the second trimester, and 35/65 (53.8%; 95% CI: 41.7–66.0%) in the third trimester. No congenital toxoplasmosis was recorded in the group of periconceptional infections: 0/12 (0%; 95% CI: 0.0–26.6%) (Figure 1).

**Figure 2:**
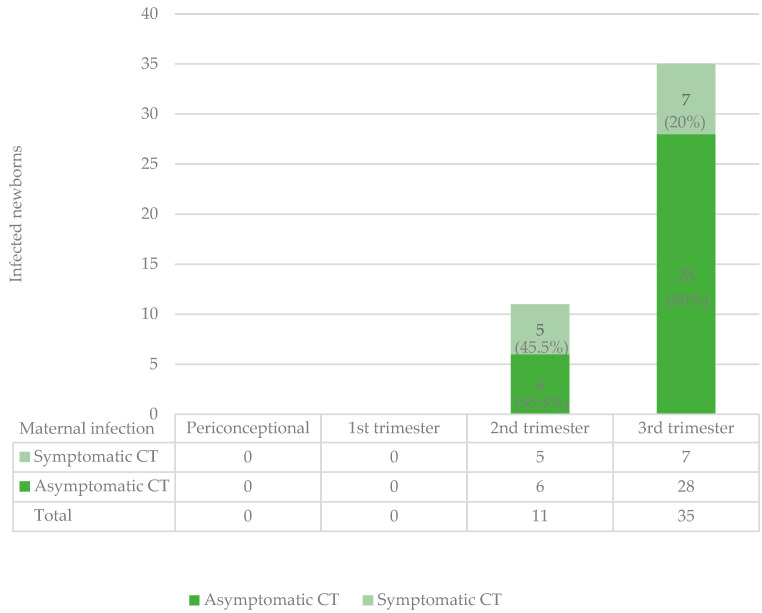
Symptomatic and asymptomatic infected newborns in relation to time of maternal infection.

Figure 2 synthesizes data concerning the group of infected newborns in terms of symptomatic or asymptomatic infection, according to the time of maternal infection; the rate of symptomatic newborns was 5/11 (45.5%; 95% CI: 16.0–74.9%) in the second-trimester SCs and 7/35 (20%; 95% CI: 6.7–33.3%) in the third-trimester ones.

Among the infected newborns, there were 12 cases with clinical manifestation (26.1%; 95% CI: 13.4–38.8%), including 1 case of stillbirth (2.2%; 95% CI: 0.0–6.4%) and 11 symptomatic neonates (23.9%; 95% CI: 11.6–36.2%). Of these symptomatic cases, five (10.9%; 95% CI: 1.9–19.9%) were from maternal infection in the second trimester and seven (15.2%; 95% CI: 4.8–25.6%) in the third trimester. Among the mothers of children with symptomatic congenital infection, 4/12 (33.3%; 95% CI: 6.7–60.0%) were born abroad, and 2/12 were not treated (16.7%; 95% CI: 0–37.8%), 11/12 (91.7%; 95% CI 76.0–100.0%) and experienced a delay of 3 weeks or more in treatment following the time of infection. Only 2/12 (16.7%; 95% CI: 0–37.8%) were treated with pyrimethamine-sulfadiazine.

We found a difference (although not statistically significant) in the number of symptomatic infected newborns between treated (10/182, 5.5%; 95% CI: 2.2–8.8%) and untreated women (2/10, 20%; 95% CI: 4.8–44.8%).

Regarding symptoms, four newborns exhibited ocular pathology (8.7%; 95% CI: 0.6–16.8%), six had cerebral involvement (13.0%; 95% CI: 3.3–22.8%), and one showed both ocular and cerebral localizations (2.2%; 95% CI: -2.0–6.4%). All the infected newborns were treated for one year with pyrimethamine-sulfadiazine plus folinic acid.

## 4. Discussion

According to the literature, the overall transmission rate from mother to fetus increases with gestational age, with no CT in the group of periconceptional infection and SC in the first trimester [1,2,3,4,5,6,14].

The transmission rates per trimester found in our cohort are lower than those reported in the literature: 0% (95% CI: 0.0–11.9%) in the first trimester, 12.5% (95% CI: 5.6–19.4%) in the second trimester, and 53.8% (95% CI: 41.7–66.0%) in the third trimester, compared to 2.2%, 23%, and 56%, respectively [3].

One explanation for this difference could be the effectiveness of monthly screening during pregnancy, careful counselling, and women’s adherence to therapy. It could be useful, in a further prospective study, to record adherence to monthly screening. However, this low transmission rate could be subject to some biases. Indeed, our population was numerically smaller than the cohort mentioned as a comparator [3].

Regarding the proportion of newborns with and without symptoms according to gestational age, our findings are in line with previous studies [3,14]. In the group of infected newborns, the percentage of symptomatic newborns was 45.5% (95% CI: 16.0–74.9%) in second-trimester SCs and only 20% (95% CI: 6.7–33.3%) in third-trimester SCs. The overall percentage of symptomatic newborns (26.1%; 95% CI: 13.4–38.8%) corresponds to previous studies [3,14]. Mothers whose children exhibited clinical signs had specific characteristics: they may have been infected abroad by more virulent strains (33.3%; 95% CI: 6.7–60.0%) and they had not promptly received treatment (16.7%; 95% CI: 0.0–37.8%). This could explain the high number of symptomatic children. A positive effect of maternal treatment during pregnancy on the risk of symptomatic disease was observed, even if not statistically significant.

Spiramycin therapy was well tolerated: only 3 out of 223 (1.3%; 95% CI: 0–2.9%) pregnant women discontinued it due to side effects. In patients treated with pyrimethamine-sulfadiazine, a higher percentage of discontinuation due to adverse effects was observed: 9 out of 51 (17.6%; 95% CI 7.2–28.1%) with the reintroduction of spiramycin therapy. These data confirm the evidence regarding poor tolerability and higher toxicity of pyrimethamine-sulfadiazine therapy [7,8,9,10].

Amniocentesis, performed at referral centres such as IRCCS Policlinico San Matteo in Pavia and Spedali Civili in Brescia, proved to be a valuable, safe, and reliable diagnostic tool. No complications or fetal losses were observed. In 5 out of 83 cases (6.0%; 95% CI: 0.9–11.1%) of positive amniocentesis, serological data and neonatal follow-up confirmed the diagnosis of congenital toxoplasmosis; meanwhile, negative infant serology at one year of age excluded the diagnosis of CT after a negative amniocentesis result. We therefore conclude that in our case series, we had no false positive or false negative results (sensitivity and specificity of 100%). It is important to rely on different techniques in the diagnosis of *T. gondii* infection, indirectly through serological tests or directly using molecular methods. The prenatal diagnosis of CT relies on the detection of parasitic DNA in amniotic fluid. Given the high sensitivity and specificity of this method, a negative result can reassure the patient, and a positive one allows us to adapt therapeutic and medical care in fetuses and newborns [11,12,13].

Another significant finding is the low number of ToPs: only one patient (0.4%; 95% CI: 0.0–1.2%) in our cohort, with a very early infection (7th gestational week) requested it. The percentage of ToP reported in the literature, in another Italian cohort, is way higher (2.8%) [15]. It would be interesting, in a future study, to evaluate the association between screening, prompt counselling based on a multidisciplinary approach, and reduction in requests for ToP.

There are some limitations of this study: (1) we could not analyze the correlation between the timing of therapy administration and the percentage of maternal–fetal transmission; (2) we lost around one quarter of newborns to follow-up; and (3) the percentage of symptomatic CT could be larger because infected newborns could present symptoms several years after CT diagnosis even if previously considered asymptomatic.

In conclusion, the results obtained are in line with the fetal risk reported by previous studies for *T. gondii* infection during pregnancy, but at a lower percentage, probably due to early treatment after SC evidence. Screening during pregnancy, comprehensive counselling, and a multidisciplinary approach for the patient are key in the diagnostic–therapeutic management of these pregnancies.

## Figures and Tables

**Table 1:** Demographic, anamnestic, and clinical data concerning pregnant women and newborns.

**Maternal age, yr, median (IQR)**	30.5 (27–34)
**Geographical origin, n (%)**	
Italy	177 (71.6; 95% CI 66.0–77.3%)
Foreign	58 (23.5; 95% CI 18.2–28.8%)
Unknown	12 (4.9; 95% CI 2.2–7.5%)
**Seroconversion trimester, n (%)**	
First (1–12 w)	38 (16.2; 95% CI 11.5–20.9%)
Second (13- 27 w)	122 (51.9; 95% CI 45.5–58.3%)
Third (28–40 w)	75 (31.9; 95% CI 26.0–37.9%)
**Termination of pregnancy, n (%)**	1/247 (0.4; 95% CI 0.0–1.2%)
**Maternal treatment, n (%)**	
Spiramycin	223 (90.3; 95% CI 86.6–94.0%)
Pyrimethamine-Sulfadiazine *	51 (20.6; 95% CI 15.6–25.7%)
No treatment	6 (2.4; 95% CI 0.5–4.3%)
Unknown	4 (1.6; 95% CI 0.0–3.2%)
**Treatment delay, wks (median)**	5 (0–15)
**Amniocentesis, n (%)**	83 (33.6; 95% CI 27.7–39.5%)
Positive	5/83 (6.1; 95% CI 0.9–11.1%)
Negative	78/83 (93.9; 95% CI 88.9–99.1%)
**Lost to follow-up newborns, n (%)**	56/249 (22.5; 95% CI 17.3–27.7%)
**Infected newborns, n (%)**	46/193 (23.8; 95% CI 17.8–29.8%)
**Clinically apparent CT, n (%)**	12/46 (26.1; 95% CI 13.4–38.8%)

* Both first therapeutic choices (seroconversions confirmed in late gestational age) and cases of therapeutic shift from spiramycin to pyrimethamine-sulfadiazine (positive amniocentesis) are included.

## Data Availability

The data that support the findings of this study are available on request from the corresponding author. The data are not publicly available due to privacy or ethical restrictions.
